# Active appendiceal bleeding recorded by choledochoscopy

**DOI:** 10.1055/a-2729-2890

**Published:** 2025-11-10

**Authors:** Chenyang Jiao, Cuixia Liu, Yi Gao, Long Yang, Yiwei Fu

**Affiliations:** 1 372209 Taizhou Clinical Medical School of Nanjing Medical University, Taizhou, China; 2 372209 Department of General Surgery, Taizhou Clinical Medical School of Nanjing Medical University, Taizhou, China

A 34-year-old man presented with painless hematochezia for 3 days. Colonoscopy performed at a secondary hospital was advanced to the terminal ileum, where a small amount of fresh blood was observed. The patient was initially diagnosed with small intestinal bleeding. On day 4 of illness, hematochezia persisted, and he was referred to our institution. Computed tomography angiography performed on admission, and no contrast medium was observed within the appendix on plain CT ( Fig. 1 **a** ); punctate contrast enhancement was noted during the arterial phase ( Fig. 1 **b** ), with further accumulation in the venous phase ( Fig. 1 **c** ). However, due to the extreme rarity of this finding, the intraluminal contrast within the appendix was not recognized during the initial image interpretation. On day 5, the patient continued to pass small amounts of bloody stool. On day 6, single-balloon enteroscopy was performed. On advancing the scope into the distal ileum, approximately 40 cm proximal to the ileocecal valve, yellowish intestinal fluid was encountered. Upon withdrawal to the cecum, active blood flow was observed emanating from the appendiceal orifice. A colonoscope was introduced into the cecum, and a choledochoscope was advanced through the biopsy channel. Bleeding from an appendiceal ulcer is not continuous but rather manifests as sporadic, pulsatile bleeding or oozing ( Video 1 ). Laparoscopic exploration revealed the appearance of the appendix is basically normal. Appendectomy was performed, and gross examination of the resected specimen demonstrated an ulcer located in the mid-portion of the appendix. Upon gross examination after appendiceal resection, an ulcer within the appendix was identified ( Fig. 2 ). The patient experienced no further bleeding after the surgery.

**Fig. 1:**
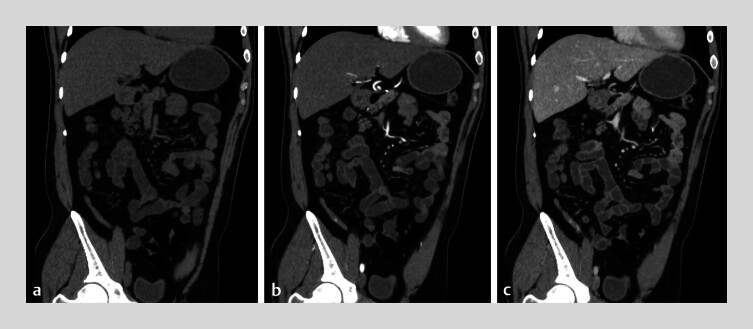
CT images of appendiceal hemorrhage. **a** CT plain scan of the appendix. **b** Arterial phase of the appendix. **c** Venous phase of the appendix.

CT scans and colonoscopy observed active bleeding in the appendix, and the insertion of a choledochoscopy into the appendiceal lumen revealed mucosal ulcers with active bleeding in the appendix.Video 1

**Fig. 2:**
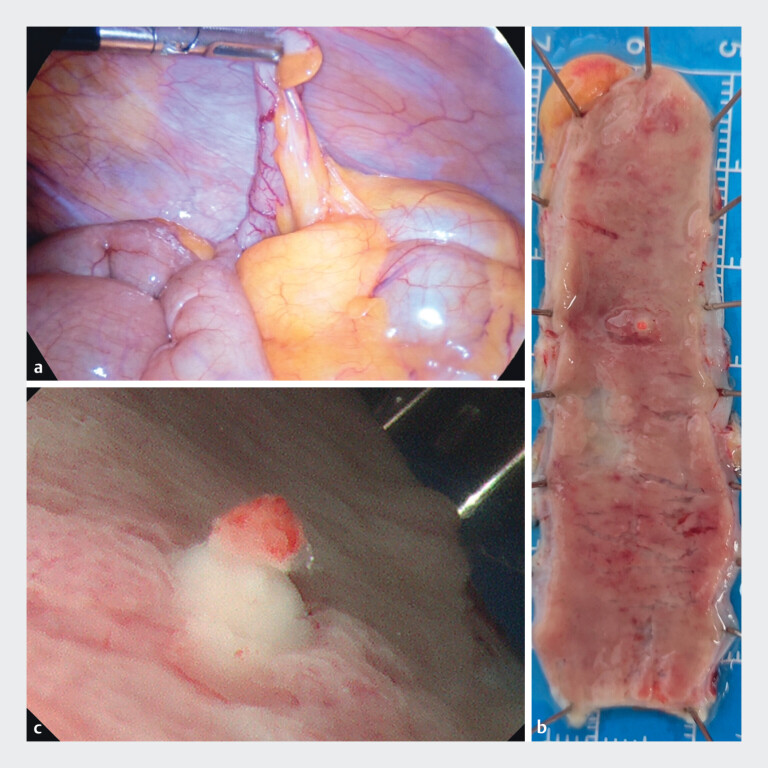
Laparoscopic and postoperative images of the appendix. **a** Laparoscopic exploration of the appendix. **b** Mucosal ulcer of the appendix. **c** Close-up observation of the appendiceal ulcer.

Appendiceal hemorrhage is a rare condition, predominantly observed in males, typically presenting with painless hematochezia 1 2 3 . There have been reports of patients with recurrent unexplained gastrointestinal bleeding who underwent multiple endoscopic examinations without identifying the bleeding source, ultimately being diagnosed with appendiceal bleeding 4 . This case represents the first reported instance of active appendiceal bleeding diagnosed and documented via cholangioscopy, providing direct evidence for the confirmation of appendiceal bleeding. Bleeding from an appendiceal ulcer is not continuous but rather manifests as sporadic, pulsatile bleeding or oozing. Choledochoscopy has the potential to become the gold standard for diagnosing appendiceal bleeding.

Endoscopy_UCTN_Code_TTT_1AQ_2AZ

## Notes

Endoscopy E-Videos https://eref.thieme.de/e-videos
*E-Videos* is an open access online section of the journal *Endoscopy* , reporting on interesting cases and new techniques in gastroenterological endoscopy. All papers include a high-quality video and are published with a Creative Commons CC-BY license. Endoscopy E-Videos qualify for HINARI discounts and waivers and eligibility is automatically checked during the submission process. We grant 100% waivers to articles whose corresponding authors are based in Group A countries and 50% waivers to those who are based in Group B countries as classified by Research4Life (see: https://www.research4life.org/access/eligibility/ ). This section has its own submission website at https://mc.manuscriptcentral.com/e-videos .
